# Peripheral Resistance Baroreflex During Incremental Bicycle Ergometer Exercise: Characterization and Correlation With Cardiac Baroreflex

**DOI:** 10.3389/fphys.2018.00688

**Published:** 2018-06-05

**Authors:** Alberto Porta, Vlasta Bari, Beatrice De Maria, Beatrice Cairo, Emanuele Vaini, Mara Malacarne, Massimo Pagani, Daniela Lucini

**Affiliations:** ^1^Department of Biomedical Sciences for Health, University of Milan, Milan, Italy; ^2^Department of Cardiothoracic, Vascular Anesthesia and Intensive Care, IRCCS Policlinico San Donato, Milan, Italy; ^3^IRCCS, Istituti Clinici Scientifici Maugeri, Milan, Italy; ^4^Dipartimento di Biotecnologie Mediche e Medicina Traslazionale, University of Milan, Milan, Italy; ^5^Exercise Medicine Unit, Humanitas Clinical and Research Center, Milan, Italy

**Keywords:** heart rate variability, laser Doppler skin blood flow, peripheral resistances, vasomotion, autonomic nervous system, cardiovascular control, sport medicine, rehabilitation

## Abstract

The arm of the baroreflex (BR) controlling peripheral resistances (PR), labeled as BR of PR (prBR), was characterized through an extension of the cardiac BR (cBR) sequence analysis. The method exploits recordings of skin blood flow (SBF) from the palm of the non-dominant hand via a laser Doppler flowmeter and of arterial pressure (AP) from the middle finger of the same hand via a plethysmographic device. PR was estimated beat-by-beat as the ratio of mean AP to mean SBF computed over the same heart period (HP). Peripheral resistances-diastolic arterial pressure (PR-DAP) sequences featuring simultaneous increases of PR and decreases of diastolic AP (DAP) or *vice versa* were identified and the slope of the regression line in the (DAP, PR) plane was taken as an estimate of prBR sensitivity (BRS_prBR_). The percentage of prBR sequences (SEQ%_prBR_) was taken as a measure of prBR involvement and the prBR effectiveness index (EI_prBR_) was computed as the fraction of DAP sequences capable to drive antiparallel PR variations. Analogous markers were computed over cBR from HP and systolic AP (SAP) variability [i.e., cBR sensitivity (BRS_cBR_), percentage of cBR sequences (SEQ%_cBR_)_,_ and effectiveness index of the cBR (EI_cBR_)]. prBR and cBR were typified during incremental light-to-moderate bicycle ergometer exercise at 10, 20, and 30% of the maximum effort in 16 healthy subjects (aged from 22 to 58 years, six males). We found that: (i) BRS_cBR_ decreased gradually with the challenge, while BRS_prBR_ declined only at the heaviest workload; (ii) SEQ%_cBR_ decreased solely at the lightest workload, while the decline of SEQ%_prBR_ was significant regardless of the intensity of the challenge; (iii) EI_prBR_ and EI_cBR_ were not affected by exercise; (iv) after pooling together all the data regardless of the experimental conditions, BRS_prBR_ and BRS_cBR_ were uncorrelated, while SEQ%_cBR_ and SEQ%_prBR_ as well as EI_cBR_ and EI_prBR_, were significantly and positively correlated; (v) when the correlation between SEQ%_cBR_ and SEQ%_prBR_ and between EI_cBR_ and EI_prBR_ was assessed separately in each experimental condition, it was not systematically detected. This study suggests that prBR characterization provides information complementary to cBR that might be fruitfully exploited to improve patients’ risk stratification.

## Introduction

Baroreflex (BR) is a composite reflex regulating several cardiovascular variables, comprising heart period (HP) (Smyth et al., 1969; Laude et al., 2004), sympathetic activity (Sundlof and Wallin, 1978; Rudas et al., 1999; Kienbaum et al., 2001; Barbic et al., 2015; Marchi et al., 2016), peripheral resistances (PR) (Borgers et al., 2014; Reyes del Paso et al., 2017), parameters associated with cardiac contractility (Reyes del Paso et al., 2017), and stroke volume (Casadei et al., 1992; Yasumasu et al., 2005; Vaschillo et al., 2012; Reyes del Paso et al., 2017), in response to arterial pressure (AP) variations (Robertson et al., 2012). The various arms of the BR are more and more frequently described simultaneously because it has been suggested that the information derived from monitoring different BR components is not fully redundant and, conversely, their simultaneous assessment might provide a more adequate and complete picture of the complexity of the cardiovascular reflex regulation (Dutoit et al., 2010; Borgers et al., 2014; Taylor et al., 2015; Marchi et al., 2016; Porta et al., 2017b; Reyes del Paso et al., 2017). The cardiac BR (cBR), namely the arm of the BR inducing HP changes parallel to those observed in AP (Smyth et al., 1969), is commonly studied in physiological, closed loop, conditions from short recordings (i.e., 5 min) of spontaneous variability of HP and systolic AP (SAP) (Laude et al., 2004). The evaluation of cBR sensitivity, quantifying the amplitude of the HP response per unit variation of SAP (Laude et al., 2004), provided clinical information useful to stratify the risk in heart failure and myocardial infarction populations (Barthel et al., 2012; Pinna et al., 2017). The set of parameters helpful for the cBR description is not limited to the cBR sensitivity but it includes also parameters evaluating the degree of cBR involvement and its effectiveness (Bertinieri et al., 1985; Di Rienzo et al., 2001; Porta et al., 2017b).

Since cBR is mainly under vagal control being cBR sensitivity depressed in situations of sympathetic activation (Parlow et al., 1995; Marchi et al., 2016; De Maria et al., 2018), BR characterization is commonly complemented by the characterization of BR arms more under sympathetic control and more directly involved in governing peripheral vasoconstriction/vasodilatation. As a consequence, the sympathetic BR (sBR), namely the arm of the BR inducing tonic changes of sympathetic activity antiparallel to those observed in diastolic AP (DAP), is also actively studied (Sundlof and Wallin, 1978). However, sBR characterization based on physiological variability is not fully non-invasive like that of cBR: as a matter of fact, sBR requires the invasive recording of sympathetic activity, usually obtained in humans via microneurographic technique by placing an electrode in the peroneal nerve at the fibular head (Sundlof and Wallin, 1978). To circumvent the involvedness of the microneurographic technique and its invasiveness, sBR characterization has been surrogated via the assessment of BR of PR (prBR) (Borgers et al., 2014; Reyes del Paso et al., 2017) exploiting the direct link between sympathetic activity and PR (Charkoudian et al., 2005). Usually, the beat-to-beat variability of PR exploited for the prBR characterization is estimated from the analysis of AP curve (Borgers et al., 2014) or via impedance cardiography (Reyes del Paso et al., 2017).

This study proposes an alternative approach to the characterization of prBR requiring the continuous monitoring of skin blood flow (SBF) and AP carried out, respectively, via a laser Doppler probe located at the palm of the hand of the non-dominant arm and via a plethysmographic cuff positioned at the middle finger of the same hand. The computation of the PR on a beat-to-beat basis is carried out as the ratio of the mean AP (MAP) to mean SBF (MSBF) over the same cardiac cycle (Porta et al., 1995). cBR sequence method was adapted for characterization of the antiparallel patterns involving PR and DAP variations induced by incremental light-to-moderate bicycle ergometer exercise challenging both cBR and prBR as a consequence of the modifications of AP (Lucini et al., 1997; Lucini et al., 2004). cBR was typified simultaneously to prBR via BR sequence analysis and the degree of redundancy of the characterization of prBR and cBR was assessed by computing the degree of association of analogous prBR and cBR parameters (Bertinieri et al., 1985; Di Rienzo et al., 2001; Marchi et al., 2016; Porta et al., 2017a). Preliminary data were presented during the 39th Annual International Conference of the Engineering in Medicine and Biology Society (Porta et al., 2017a).

## Materials and Methods

### Characterization of cBR

**FIGURE 1:**
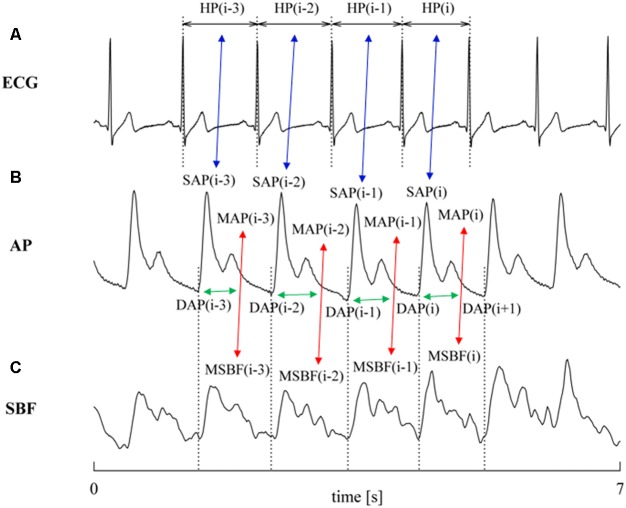
The conventions for the construction of HP, SAP, DAP, and PR variability series from ECG **(A)**, AP **(B)**, and SBF **(C)** are shown. MAP and MSBF are computed within two consecutive diastolic values and their combination, denoted by red double arrows, allows the computation of PR. The associations between HP and SAP values at the same cardiac beat and between PR and DAP values at the same cardiac beat are made evident via blue and green double arrows, respectively. Association might be varied according with the selected value of *τ*_HP-SAP_ and of *τ*_PR-DAP_ (here *τ*_HP-SAP_ = *τ*_PR-DAP_ = 0). The dotted lines identify the positions of the QRS complex on the ECG trace and the diastolic values on the AP trace.

The characterization of cBR was carried out via the cBR sequence method (Bertinieri et al., 1985) as implemented in Porta et al. (2000). This technique relies on the search for joint patterns of HP and SAP featuring parallel variations of HP and SAP usually indicated as +/+ and -/- sequences according to the sign of variations. The HP and SAP sequences forming the joint HP-SAP +/+ and -/- patterns were composed by four consecutive HP and SAP values presenting three positive or negative variations (Porta et al., 2013). **Figure 1** shows an example of joint HP-SAP pattern formed by associating four values of SAP with correspondent values of HP (**Figure 1A**, blue double arrows). The method tests the cBR origin of this joint HP-SAP pattern by checking whether it belongs to the +/+ or -/- category. A spontaneous HP-SAP +/+ and -/- pattern was considered to be of cBR origin regardless of the absolute magnitude of total, or partial, HP and SAP variations and degree of HP-SAP association (Porta et al., 2013). The cBR patterns were usually represented in the [SAP(*i*), HP(*i* + *τ*_HP-SAP_)] plane, where *i* is the progressive cardiac beat counter and *τ*_HP-SAP_ represents the latency of the cBR expressed in cardiac beats. In the [SAP(*i*), HP(*i* + *τ*_HP-SAP_)] plane the cBR patterns were individually fitted with a regression line and the *r* was utilized as a measure of the degree of HP-SAP association. This procedure requires one to choose the *τ*_HP-SAP_. Therefore, in agreement with the site of AP measurement (i.e., at finger level), the measurement conventions taking SAP(*i*) within HP(*i*) [i.e., SAP(*i*) occurred when HP(*i*) was not ended yet], the shortest latency of cBR (i.e., 0.24 s) (Eckberg, 1976; Baselli et al., 1994), the time necessary to see the crossing to zero of the HP response to one neck suction stimulation (i.e., 3 s) (Baskerville et al., 1979), and the type of protocol inducing a tachycardic response, the optimal *τ*_HP-SAP_, *τ*_HP-SAP_°, was chosen in the range from 0 to 5 beats as the value of *τ*_HP-SAP_ making the HP-SAP *r* closer to +1. The slopes of the regression lines computed in the [SAP(*i*), HP(*i* + *τ*_HP-SAP_°)] plane were averaged over all cBR patterns. This average was taken as an estimate of the BR sensitivity of the cBR arm (BRS_cBR_). By definition BRS_cBR_ was positive or equal to zero. After linear fitting the cBR patterns in the [SAP(*i*), HP(*i* + *τ*_HP-SAP_°)] plane took the form of ramps with positive slope. The percentage of cBR patterns with respect to all possible joint HP-SAP patterns of length 4 beats was computed as well and indicated as SEQ%_cBR_. SEQ%_cBR_ ranges between 0 and 100, where 0 indicates the absence of cBR patterns, while 100 indicates that all possible HP-SAP patterns are of cBR origin. SEQ%_cBR_ was taken as a measure of the degree of cBR involvement. The fraction of SAP positive or negative sequences capable of evoking parallel HP variations was taken as cBR effectiveness index (EI_cBR_) (Di Rienzo et al., 2001). It ranges between 0 and 1 where 0 indicates that none of the SAP positive or negative sequences drove parallel HP changes (i.e., the efficacy of the cBR was null) and 1 indicates that all the observed SAP positive or negative sequences drove parallel HP variations (i.e., the efficacy of the cBR was maximum).

### Characterization of prBR

The characterization of prBR was carried out in analogy with the method utilized to typify sBR from the variability of sympathetic neural discharge in humans (Marchi et al., 2016). The technique is based on the same principle as cBR sequence analysis, namely the search of joint patterns over spontaneous fluctuations of two physiological variables. For the characterization of prBR we considered the physiological variability of PR and DAP. We searched for joint PR-DAP patterns featuring antiparallel variations of PR and DAP, indicated as +/- and -/+ sequences according to the sign of variations. The PR and DAP sequences forming the joint PR-DAP +/- and -/+ patterns were composed by four consecutive PR and DAP values presenting three positive variations of PR and three negative variations of DAP or *vice versa*, respectively (Marchi et al., 2016). **Figure 1** shows an example of joint PR-DAP pattern formed by associating four values of DAP with correspondent values of PR (**Figure 1B**, green double arrows), obtained from MAP and MSBF computed over the same cardiac cycle (**Figures 1B,C**, red double arrows). The method tests the prBR origin of this joint PR-DAP pattern by checking whether it belongs to the +/- or -/+ categories. A spontaneous PR-DAP +/- and -/+ pattern was considered to be of prBR origin regardless of the absolute magnitude of total, or partial, PR and DAP variations and the degree of PR-DAP association. The prBR patterns could be represented in the [DAP(*i*), PR(*i* + *τ*_PR-DAP_)] plane, where *τ*_PR-DAP_ is the latency of the prBR expressed in cardiac beats. In the [DAP(*i*), PR(*i* + *τ*_PR-DAP_)] plane the prBR patterns were individually fitted with a regression line and the *r* was utilized as a measure of the degree of PR-DAP association. This procedure requires one to choose *τ*_PR-DAP_. Therefore, in agreement with site of AP measurement (i.e., at finger level), conventions of time series [i.e., DAP(*i*) is taken before SAP(*i*)], the shortest and the longest latency of sBR (i.e., 1.16 and 1.45 s, respectively) (Sundlof and Wallin, 1978; Diedrich et al., 2009), and the type of protocol inducing a tachycardic response, the optimal *τ*_PR-DAP_, *τ*_PR-DAP_°, was chosen in the range from 0 to 5 beats as the value of *τ*_PR-DAP_ making the PR-DAP *r* closer to -1. The slopes of the regression lines computed in the [DAP(*i*), PR(*i* + *τ*_PR-DAP_°)] plane were averaged over all prBR patterns. This average was taken as an estimate of the BR sensitivity of the prBR arm (BRS_prBR_). By definition BRS_prBR_ was negative or equal to 0. After linear fitting the prBR patterns in the [DAP(*i*), PR(*i* + *τ*_PR-DAP_°)] plane took the form of ramps with negative slope. The percentage of prBR patterns with respect to all possible PR-DAP patterns of length 4 beats was computed as well and indicated as SEQ%_prBR_. SEQ%_prBR_ ranges between 0 and 100, where 0 indicates the absence of prBR patterns, while 100 indicates that all possible PR-DAP patterns are of prBR origin. SEQ%_prBR_ was taken as a measure of the degree of prBR involvement. The fraction of DAP positive or negative sequences capable of evoking antiparallel PR variations was taken as prBR effectiveness index (EI_prBR_). It ranges between 0 and 1 where 0 indicates that none of the DAP positive or negative sequences drove antiparallel PR changes (i.e., the efficacy of the prBR was null) and 1 indicates that all the observed DAP positive or negative sequences caused antiparallel PR variations (i.e., the efficacy of the prBR was maximum).

## Experimental Protocol and Data Analysis

### Ethics Statement

Data belong to an historical database designed to study the autonomic control during graded dynamical exercise in humans via the analysis of spontaneous variability of systemic and peripheral physiological variables (Porta et al., 1995, 1999; Lucini et al., 1997, 2004). A full description of the experimental protocol and population is reported elsewhere (Porta et al., 1995, 1999; Lucini et al., 1997, 2004). The study was carried out in keeping with the Declaration of Helsinki for medical research involving human subjects and was approved by the ethical review board of the “Luigi Sacco” Hospital, Milan, Italy. Written signed informed consent was obtained from all subjects.

### Experimental Protocol

Briefly, we studied 16 healthy humans (aged from 22 to 58 years, 40 ± 12 years, mean ± standard deviation, six males). ECG from lead II (ECG Bioamplifier, Marazza, Monza, Italy), finger plethysmographic AP (Finapres Monitor, Ohmeda, Englewood, CO, United States) and SBF assessed via a laser Doppler probe (Multichannel Laser Doppler System, PeriFlux 4001 Master, Perimed, Pärfälla, Sweden) were acquired at 300 Hz. The SBF was recorded from the palm of the hand of the non-dominant arm. AP was taken from the middle finger of the same hand. The laser Doppler flowmeter provided an estimate of the SBF velocity based on the Doppler shift of the reflected red light due to the movement of the blood cells in the microvasculature below the probe. Standard and invariable geometrical parameters were utilized to convert the velocity into flow in the considered region. Since these parameters were not available individually, the SBF was expressed in arbitrary laser Doppler shift unit (LDsu). The measure was proportional to flow under the hypotheses that the assigned parameters matched the subject’s characteristics and remained constant within the experiment. The auto-calibration procedure of the AP device was switched off after the first automatic calibration. The AP signal was cross-calibrated using a measure provided by a sphygmomanometer before the onset of the recording session. The subjects were positioned in a recumbent position on the bicycle ergometer (Lode, Groningen, Netherlands). A period of 10 min was allowed for stabilization. Then, signals were recorded for 10 min at rest (R), for 5 min during electronically braked exercise at 10 (E10), 20 (E20), and 30% (E30) of the maximum load identified individually during a session of incremental exercise until exhaustion carried out the day before, and for 10 min during recovery (RC). During exercise pedaling rate was maintained at 60 revolutions per minute.

### Extraction of the Beat-to-Beat Variability Series

Heart period was measured as the temporal distance between two consecutive QRS complexes detected on the ECG (**Figure 1A**) using a method based on a threshold on the first derivative (Porta et al., 1998). The *i*th SAP [i.e., SAP(*i*)] was the maximum value of AP within the *i*th HP [i.e., HP(*i*)] (**Figure 1B**). The *i*th DAP [i.e., DAP(*i*)] was the AP minimum found before SAP(*i*) (**Figure 1B**). The *i*th MAP [i.e., MAP(*i*)] was computed as the integral of AP between the occurrence of the *i*th and (*i* + 1)th DAP values divided by the current inter-diastolic time interval (**Figure 1B**). The same fiducial points were utilized for the computation of the *i*th MSBF [i.e., MSBF(*i*)] over the SBF signal (**Figure 1B**). The *i*th PR value was obtained as the ratio of MAP(*i*) to the MSBF(*i*) (Porta et al., 1995). In agreement with short-term analysis of cardiovascular control (Task Force of the European Society of Cardiology and the North American Society of Pacing and Electrophysiology, 1996), sequences of 256 consecutive measures were randomly selected inside R, E10, E20, E30, and RC periods, respectively. The mean of HP, SAP, DAP, and PR series were computed over the original series and expressed as ms, mmHg, mmHg, and mmHg⋅LDsu^-1^ respectively. The variances of HP, SAP, DAP, and PR series were calculated over the linearly detrended series and expressed as ms^2^, mmHg^2^, mmHg^2^, and mmHg^2^⋅LDsu^-2^ respectively.

### Statistical Analysis

One-way repeated measures analysis of variance (Dunnett’s test for multiple comparisons), or Friedman one-way repeated measures analysis of variance on ranks when appropriate (Dunnett’s test for multiple comparisons), was applied to test the significance of the changes of cBR and prBR markers versus R. Linear regression analysis was carried out to quantify the degree of association between analogous indexes describing cBR and prBR. The Pearson product moment *r* was computed and the null hypothesis of a slope equal to zero (i.e., no linear relationship) was tested after pooling together all the subjects regardless of the experimental condition. If the null hypothesis of homoscedasticity was rejected in the case of either of the variables or both, the result of linear regression analysis was checked after log-transforming the absolute values of the variable that did not pass the test for homogeneity of variance. Linear regression analysis was repeated in every experimental conditions as well. Statistical analysis was carried out using a commercial statistical program (Sigmaplot, ver.11.0, Systat Software, San Jose, CA, United States). A *p* < 0.05 was always considered as significant.

## Results

**FIGURE 2:**
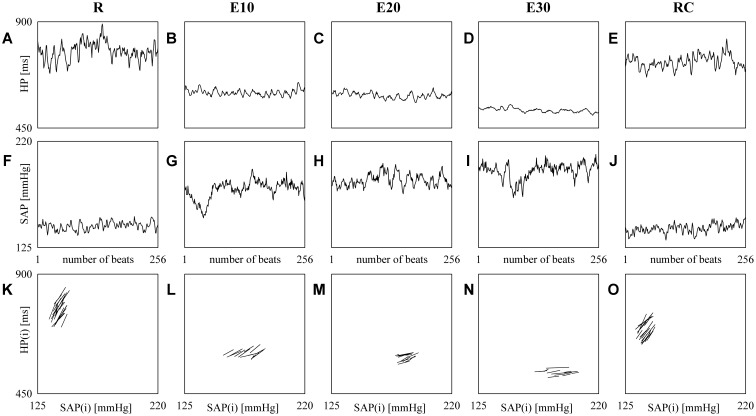
The line plots show examples of HP (top panels) and SAP (middle panels) beat-to-beat variability recorded at R **(A,F)**, during E10 **(B,G)**, E20 **(C,H)**, E30 **(D,I)**, and RC **(E,J)**. The line plots (bottom panels) show the linearly-interpolated spontaneous HP-SAP +/+ and –/– patterns in the [SAP(*i*), HP(*i* + *τ*_HP-SAP_°)] plane at R **(K)**, during E10 **(L)**, during E20 **(M)**, during E30 **(N)**, and during RC **(O)** extracted from the HP and SAP variability series depicted, respectively, in **(A–J)**.

**Figure 2** shows examples of HP (top panels) and SAP (middle panels) series recorded from the same subject at R (**Figures 2A,F**), during E10 (**Figures 2B,G**), E20 (**Figures 2C,H**), E30 (**Figures 2D,I**) and RC (**Figures 2E,J**). The HP mean and variance gradually decrease with the intensity of the exercise (**Figures 2B–D**) and return to the baseline values during RC (**Figure 2E**). SAP mean and variance show the opposite tendency (**Figures 2G–I**) and baseline values are restored during RC (**Figure 2J**). The line plots (bottom panels) show the linearly-interpolated spontaneous HP-SAP +/+ and -/- patterns in the [SAP(*i*), HP(*i* + *τ*_HP-SAP_°)] plane at R (**Figure 2K**), during E10 (**Figure 2L**), during E20 (**Figure 2M**), during E30 (**Figure 2N**) and during RC (**Figure 2O**) extracted from the HP and SAP variability series depicted, respectively, in **Figures 2A–J**. The slopes of the linear regressions are positive (**Figures 2K–O**). Moreover, at R and during RC (**Figures 2K,O**) they are steeper and their number is larger (**Figures 2K,O**) than during E10, E20, and E30 (**Figures 2L–N**): BRS_cBR_ is equal to 8.73, 2.99, 1.77, 0.79, and 5.01 ms mmHg^-1^ and SEQ%_cBR_ is equal to 7.87, 5.12, 5.91, 5.12, and 7.87 at R, during E10, E20, E30, and RC respectively.

**FIGURE 3:**
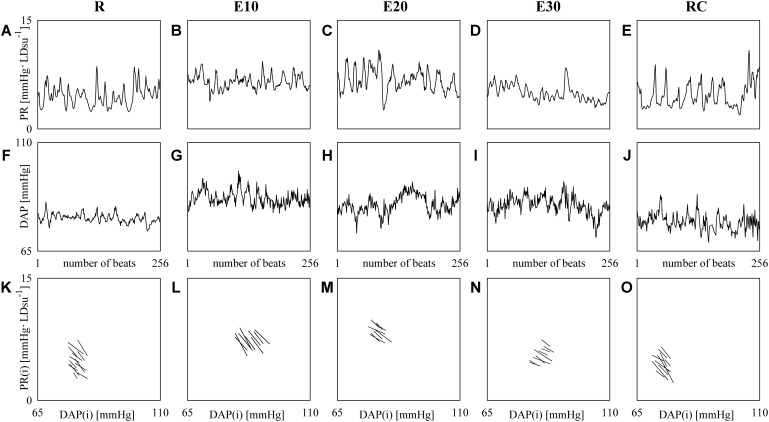
The line plots show examples of PR (top panels) and DAP (middle panels) beat-to-beat variability recorded at R **(A,F)**, during E10 **(B,G)**, E20 **(C,H)**, E30 **(D,I)**, and RC **(E,J)**. The line plots (bottom panels) show the linearly-interpolated spontaneous PR-DAP +/– and –/+ patterns in the [DAP(*i*), PR(*i + τ*_PR-DAP_°)] plane at R **(K)**, during E10 **(L)**, during E20 **(M)**, during E30 **(N)**, and during RC **(O)** extracted from the PR and DAP variability series depicted, respectively, in **(A–J)**.

**Figure 3** shows examples of PR (top panels) and DAP (middle panels) series recorded from the same subject at R (**Figures 3A,F**), during E10 (**Figures 3B,G**), E20 (**Figures 3C,H**), E30 (**Figures 3D,I**), and RC (**Figures 3E,J**). The PR mean increases and the rise is particularly evident during E10 and E20 (**Figures 3B,C**), while the variance is not significantly modified. DAP mean and variance increase with the intensity of the exercise (**Figures 3G–I**). The line plots (bottom panels) show the linearly-interpolated spontaneous PR-DAP +/- and -/+ patterns in the [DAP(*i*), PR(*i* + *τ*_PR-DAP_°)] plane at R (**Figure 3K**), during E10 (**Figure 3L**), during E20 (**Figure 3M**), during E30 (**Figure 3N**), and during RC (**Figure 3O**) extracted from the PR and DAP variability series depicted, respectively, in **Figures 3A–J**. The slopes of the linear regressions are negative (**Figures 3K–O**). The regression lines are almost flat during E30 (**Figure 3N**) and the number of prBR patterns decreases during exercise (**Figures 3L–N**): BRS_prBR_ is equal to -0.34, -0.45, -0.27, -0.19, and -0.39 LDsu^-1^ and SEQ%_prBR_ is equal to 5.12, 3.54, 3.15, 3.54, and 4.72 at R, during E10, E20, E30, and RC respectively.

Over the entire population HP mean gradually decreased with the workload (i.e., 888.44 ± 153.08, 689.33 ± 76.19, 618.77 ± 62.87, and 559.08 ± 56.40 ms at R, during E10, E20, and E30 respectively) and was restored during RC (i.e., 834.46 ± 104.97 ms). The same trend was observed in the case of HP variance (i.e., 2164.21 ± 2452.45, 736.32 ± 821.67, 313.71 ± 234.02, 133.77 ± 74.26, and 2217.21 ± 2680.64 ms^2^ at R, during E10, E20, E30, and RC respectively). SAP and DAP means increased during the dynamical exercise and returned to baseline values during RC (i.e., 126.69 ± 16.27, 148.11 ± 22.24, 148.87 ± 17.52, 159.84 ± 19.00, and 122.57 ± 11.50 mmHg at R, during E10, E20, E30, and RC respectively, and 78.31 ± 9.29, 84.93 ± 10.18, 84.13 ± 9.30, 87.42 ± 8.49, and 75.54 ± 10.21 mmHg at R, during E10, E20, E30, and RC, respectively). Same trends were observed in the case of SAP variance (i.e., 16.91 ± 10.80, 28.02 ± 25.89, 29.72 ± 19.52, and 33.85 ± 17.11 mmHg^2^ at R, during E10, E20, and E30, respectively), and DAP variance (i.e., 5.67 ± 4.66, 8.15 ± 5.67, 8.39 ± 6.08, and 9.00 ± 6.45 mmHg^2^, respectively at R, during E10, E20, and E30). Both SAP and DAP variances declined toward the basal values during RC (i.e., 25.02 ± 20.17 and 7.59 ± 7.13 mmHg^2^, respectively). PR mean increased significantly during E10 and E20 compared to R (i.e., 5.83 ± 5.51 and 4.04 ± 3.67 versus 3.27 ± 4.34 mmHg⋅LDsu^-1^), while it was similar to R during E30 and RC (i.e., 2.70 ± 2.62 and 2.02 ± 1.92 mmHg⋅LDsu^-1^). PR variance remained stable during the dynamical exercise protocol (i.e., 3.05 ± 5.20, 5.72 ± 8.56, 3.67 ± 6.62, 1.03 ± 1.94, and 1.24 ± 2.15 mmHg^2^⋅LDsu^-2^ at R, during E10, E20, E30, and RC, respectively).

**FIGURE 4:**
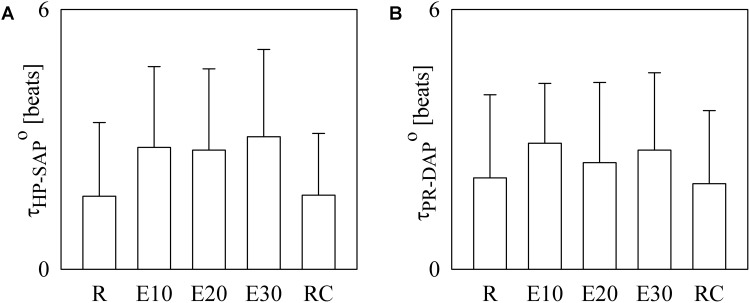
The bar graphs show *t*_HP-SAP_° **(A)** and *t*_PR-DAP_° **(B)** as estimated individually in all experimental conditions (i.e., R, E10, E20, E30, and RC). Values are given as mean plus standard deviation.

The bar graphs of **Figure 4** show the optimal *τ*_HP-SAP_, *τ*_HP-SAP_° (**Figure 4A**), and the optimal *τ*_PR-DAP_°. as estimated on a individual basis. In **Figure 4A** small values of *τ*_HP-SAP_° were more frequent at R and during RC (i.e., 1.69 ± 1.70 and 1.71 ± 1.42 beats respectively), while higher values were found during E10, E20, and E30 (i.e., 2.81 ± 1.87, 2.75 ± 1.88, and 3.06 ± 2.02 beats respectively). In **Figure 4B** *τ*_PR-DAP_° exhibited a similar course but differences between R (or RC) and exercise sessions were less evident (i.e., 2.11 ± 1.92, 2.91 ± 1.38, 2.46 ± 1.85, 2.75 ± 1.79, and 1.97 ± 1.69 beats at R, during E01, E20, E30, and RC, respectively).

**FIGURE 5:**
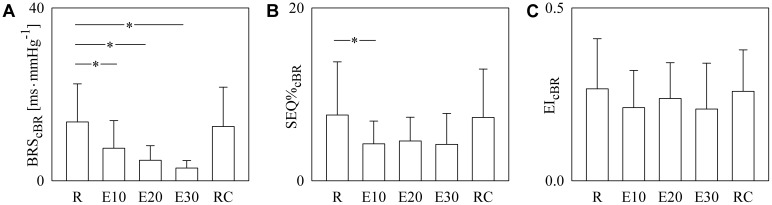
The bar graphs show BRS_cBR_ **(A)**, SEQ%_cBR_ **(B)**, and EI_cBR_ **(C)** as a function of the experimental condition (i.e., R, E10, E20, E30, and RC). Values are given as mean plus standard deviation. The symbol ^∗^ denotes *p* < 0.05 versus R.

The bar graphs of **Figure 5** show BRS_cBR_ (**Figure 5A**), SEQ%_cBR_ (**Figure 5B**), and EI_cBR_ (**Figure 5C**) as a function of the experimental condition (i.e., R, E10, E20, E30, and RC). BRS_cBR_ gradually moved toward 0 with the exercise workload (**Figure 5A**). SEQ%_cBR_ decreased during dynamical exercise as well even though the decline was significant only during E10 (**Figure 5B**). EI_cBR_ did not vary with the experimental condition (**Figure 5C**).

**FIGURE 6:**
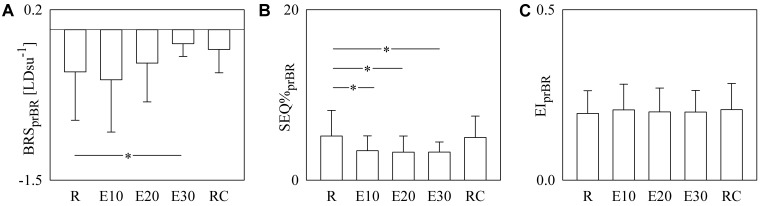
The bar graphs show BRS_prBR_ **(A)**, SEQ%_prBR_ **(B)**, and EI_prBR_ **(C)** as a function of the experimental condition (i.e., R, E10, E20, E30, and RC). Values are given as mean plus standard deviation. The symbol ^∗^ denotes *p* < 0.05 versus R.

**Figure 6** has the same structure as **Figure 5** but it shows BRS_prBR_ (**Figure 6A**), SEQ%_prBR_ (**Figure 6B**), and EI_prBR_ (**Figure 6C**) as a function of the experimental condition (i.e., R, E10, E20, E30, and RC). BRS_prBR_ tended to become less negative with the exercise workload and this tendency was significant during E30 (**Figure 6A**). SEQ%_prBR_ decreased during exercise and the decline was significant regardless of the intensity of the challenge (**Figure 6B**). EI_prBR_ was stable during the protocol (**Figure 6C**).

**FIGURE 7:**
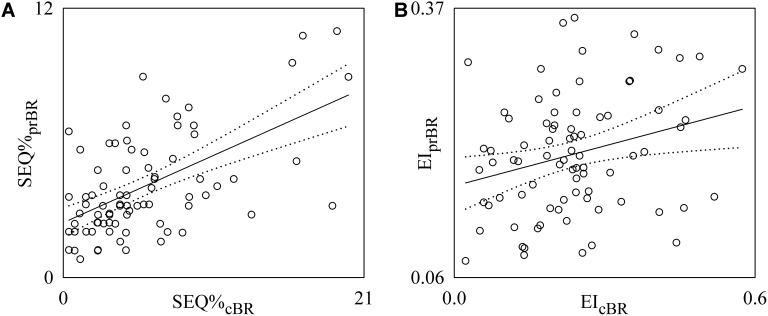
Linear regression analyses of SEQ%_prBR_ on SEQ%_cBR_ **(A)** and EI_prBR_ on EI_cBR_ **(B)** during incremental light-to-moderate bicycle ergometer exercise. Individual pairs (open circles) in the (SEQ%_cBR_, SEQ%_prBR_) and (EI_cBR_, EI_prBR_) planes are shown after pooling all subjects together regardless of the experimental condition. The linear regression (solid line) and its 95% confidence interval (dotted lines) are plotted as well if the slope of the regression line is significantly larger than zero with *p* < 0.05.

**Figure 7** shows the individual pairs (open circles) in the (SEQ%_cBR_, SEQ%_prBR_) (**Figure 7A**) and (EI_cBR_, EI_prBR_) (**Figure 7B**) planes as estimated from each subject regardless of the experimental condition. The regression lines of SEQ%_prBR_ on SEQ%_cBR_ and of EI_prBR_ on EI_cBR_ (solid line) and their 95% confidence interval (dotted lines) are plotted as well when the slopes of the regression line are significantly larger than 0. SEQ%_cBR_ was significantly and positively associated with SEQ%_prBR_ (**Figure 7A**: *r* = 0.571, *p* = 3.16 × 10^-8^). Also EI_cBR_ was significantly and positively associated with EI_prBR_ even though correlation was weaker (**Figure 7B**: *r* = 0.27, *p* = 1.47 × 10^-2^). Conversely BRS_cBR_ and BRS_prBR_ were uncorrelated (*p* = 3.67 × 10^-1^). When the linear association between SEQ%_cBR_ and SEQ%_prBR_ and between EI_cBR_ and EI_prBR_ was assessed within the same experimental condition, significant correlations were not systematically detected in any condition.

## Discussion

The main findings of this study can be summarized as follows: (i) we propose a characterization of the prBR based on the evaluation of spontaneous fluctuations of PR and DAP derived from the recording of SBF obtained from the palm of the non-dominant hand using a laser Doppler probe and plethysmographic recording of AP obtained from the middle finger of the same hand; (ii) traditional sequence analysis method utilized to characterize cBR from HP and SAP variability was adapted to typify the antiparallel relation linking positive DAP changes to negative PR variations or *vice versa*; (iii) BRS_cBR_ progressively decreased during incremental light-to-moderate bicycle ergometer exercise protocol, while BRS_prBR_ declined solely at the heaviest workload; (iv) SEQ%_prPR_ decreased significantly during exercise regardless of the intensity of the challenge, while the decline of SEQ%_cBR_ was evident solely at the lightest workload; (v) EI_prBR_ and EI_cBR_ were not affected by exercise load; (vi) after pooling together all the data regardless of the experimental condition, BRS_cBR_ and BRS_prBR_ were uncorrelated, while SEQ%_cBR_ and SEQ%_prBR_, as well as EI_cBR_ and EI_prBR_, were significantly and positively correlated; (vii) when correlation was assessed separately in each experimental condition, correlation between SEQ%_cBR_ and SEQ%_prBR_ and between EI_cBR_ and EI_prBR_ was not systematically detected in all the experimental conditions.

### Characterization of the prBR via SBF and AP Beat-to-Beat Fluctuations

The characterization of the prBR from spontaneous variations of cardiovascular variables is traditionally carried out by exploiting the variability of total PR as estimated from the analysis of the AP curve (Borgers et al., 2014) and impedance cardiography (Reyes del Paso et al., 2017). This study proposes the characterization of the prBR via the continuous monitoring of SBF and AP taken at the same hand. Since the SBF signal is recorded at the level of the palmar region, SBF is representative of the flow in a specific district and, thus, highly dependent of local factors (e.g., regional variations of the temperature) and myogenic regulations due to local variations of nitric oxide release. These factors might modify PR by altering the distribution of blood in the most superficial layers of the skin under the SBF probe and these modifications might be independent of AP changes. However, since the exercise is of light intensity, especially at the beginning of the protocol, sessions are of brief duration, and peripheral district is not directly involved in the physical exercise, the considered PR variability seems to be suitable to mirror the sympathetic activation induced by exercise and the characterization of the prBR might provide an adequate non-invasive description of the sBR inducing cutaneous vasodilatation/vasoconstriction in the palmar region.

The study proposes an extension of cBR sequence analysis (Bertinieri et al., 1985; Di Rienzo et al., 2001) for the characterization of the arm of the BR responsible for the increase of PR in response to decrease of DAP or *vice versa*. DAP was chosen instead of SAP or MAP because the strength of the DAP relation with sympathetic activity is much stronger (Sundlof and Wallin, 1978; Rudas et al., 1999; Kienbaum et al., 2001; Barbic et al., 2015; Marchi et al., 2016). The BRS_prBR_ was computed as the slope of the regression line in the [DAP(*i*), PR(*i* + *τ*_PR-DAP_)] plane over sequences featuring antiparallel PR and DAP variations regardless of whether they linked positive PR changes to negative DAP variations or *vice versa*. The latency *τ*_PR-DAP_ was optimized in a range of physiologically plausible values by selecting the value of *τ*_PR-DAP_ leading to the correlation coefficient closer to -1. In agreement with the characterization of sBR based on spontaneous sequence analysis (Marchi et al., 2016), the length of the PR-DAP patterns was kept constant and equal to 4 values (i.e., three variations) and, in agreement with (Porta et al., 2013), all prBR patterns were considered to be meaningful regardless of the absolute amplitude of the total, or partial. PR and DAP variations and degree of PR-DAP association. BRS_prBR_, SEQ%_prBR_, and EI_prBR_ were taken as estimates, respectively, of the magnitude of the PR response driven by a unit change of DAP, of the degree of prBR involvement in modifying PR, and of the efficacy of a DAP variation in producing a modification of PR.

### cBR and prBR During Incremental Light-to-Moderate Bicycle Ergometer Exercise

As expected, incremental light-to-moderate bicycle ergometer exercise induced a progressive decrease of HP and its variability and a gradual increase of SAP and DAP and their variabilities (Lucini et al., 2004). This finding has been interpreted as the consequence of the sympathetic activation and vagal withdrawal associated with the challenge (Lucini et al., 2004). Sympathetic activation is expected to produce an increase of PR given that skin microvessels of the palmar district are structures rich of autonomic innervation (Bernardi et al., 1996). Accordingly, PR increased at the lightest workloads and this result provided further support to the relevance of sympathetic activation even when exercise is of light intensity. In agreement with the decline of the magnitude of the HP variability and the rise of amplitude of SAP fluctuations, we observed a progressive decline of BRS_cBR_ (Vallais et al., 2009). The decrease of BRS_cBR_ was accompanied by a maintained effectiveness of cBR as denoted by the steady level of EI_cBR_ with the relevance of the challenge. Conversely, no gradual modification of BRS_prBR_ was detected: indeed, BRS_prBR_ declined significantly solely at the heaviest intensity of the challenge. This finding stresses that the various components of the BR operate differently: indeed, while the cBR, more under vagal control, adjusts progressively its gain in proportion to the stimulus, the gain of a BR arm more under sympathetic control (i.e., the prBR) is modified only when the intensity of the challenge is more relevant. This result might be the consequence of the high inter-subject variability of the sympathetic drive in presence of similar AP values (Charkoudian et al., 2005) and its reduction at the heaviest intensity of the challenge. Inaccuracies in the calibration of the SBF resulting from imprecise geometrical factors and erroneous assumptions, that might have increased the variability of the BRS_prBR_ estimate, are unlikely to have played a relevant role on conclusions given that these inaccuracies should be invariable within the same subject and completely accounted for by repeated measures statistical analysis. Remarkably, an index independent of the amplitude of the PR changes but accounting for the detection of PR-DAP patterns of prBR origin, such as SEQ%_prBR_, indicated that the prBR was affected by sympathetic activation induced by dynamical exercise protocol. The decline of SEQ%_prBR_ suggests a reduced involvement of prBR during the exercise and stresses the relevance of mechanisms regulating PR variations independently of DAP changes such as central commands or local myogenic controls (Bernardi et al., 1997). This consideration stresses the relevance of the proposed approach to characterize the branch of the BR targeting PR of a specific district, even in absence of a precise calibration procedure of SBF. The impact of local phenomena related to the increase of temperature, usually accompanying physical exercise and modifying PR, is expected to be minimum especially at the lightest intensity of the challenge and in district that are not directly involved in the physical exercise.

### Correlation Between cBR and prBR Functioning During Incremental Light-to-Moderate Bicycle Ergometer Exercise

BR is a composite reflex featuring several arms, all aiming at maintaining AP and buffering AP variations by targeting different physiological variables (Robertson et al., 2012). The most important consequence of considering the BR as an aggregate of components is that these arms might feature a certain degree of independence in relation to their specialized characteristic, different efferent pathways and diverse levels of integration in presence of a quote of redundancy and correlation resulting from sharing the same finalistic goal. This view was verified by all the studies that simultaneously characterized more than one arm of the BR (Dutoit et al., 2010; Borgers et al., 2014; Taylor et al., 2015; Marchi et al., 2016; Porta et al., 2017b; Reyes del Paso et al., 2017). For example, it has been recently found that cBR and sBR are significantly associated during incremental orthostatic challenge, even though the amount of correlation between markers of their sensitivities and degrees of involvement is modest (Taylor et al., 2015; Marchi et al., 2016). Even a more important disagreement was found when correlation between the functioning of cBR and sBR was assessed under pharmacological stimulation (Dutoit et al., 2010) and when the association between cBR and prBR was tested using prBR markers estimated from spontaneous variability of total PR as derived from the analysis of AP wave morphology (Borgers et al., 2014). The present study confirms this view. As a matter of fact, we found that markers of sensitivity of cBR and prBR were unrelated and indexes assessing the degree of involvement and effectiveness of cBR and prBR were significantly and positively correlated, even though a certain degree of variability about the regression line was clearly visible and, more remarkably, varied with the experimental condition. We suggest that the coherent quote among markers of different BR arms is to be considered the major contribution of mechanisms integrating the various components at central level, while the incoherent quote might provide an independent information useful to improve clinical scores of risk above and beyond traditional cBR markers (La Rovere et al., 1998). The partial uncorrelation of prBR on cBR might be related to the completely different spectral content of HP and SAP variability series compared to PR and DAP variabilities. Indeed, while HP and SAP series feature both low frequency oscillations (from 0.04 to 0.15 Hz) and high frequency fluctuations at the respiratory rate (Pagani et al., 1997), PR and DAP variability series exhibit almost exclusively low frequency rhythms with periodicities even longer than those detectable in the low frequency band in HP and SAP series (Colantuoni et al., 1984; Porta et al., 1995, 1999; Bernardi et al., 1996, 1997; Stefanovska et al., 1999). This different spectral content makes more likely that BR patterns extracted from HP and SAP series occur along time scales compatible with fast respiratory oscillations (Parlow et al., 1995; Porta et al., 2000), while BR patterns derived from PR and DAP series occur along time scales compatible with slower oscillations typical of sympathetic modulation (Marchi et al., 2016).

### Limitations of the Study and Future Developments

The hypothesis that the present characterization of prBR could be exploited as a proxy of the description of sBR needs more specific studies assuring the contemporaneous recording of sympathetic activity, e.g., via microneurographic technique from the peroneal nerve, and SBF, thus allowing the direct comparison between markers of sBR (Sundlof and Wallin, 1978; Kienbaum et al., 2001; Marchi et al., 2016) and of prBR. Moreover, future studies should test whether conclusions of the present work hold when the variability of PR estimated from a specific district is substituted with the spontaneous fluctuations of the total PR assessed from the analysis of the AP waveform morphology (Borgers et al., 2014) or derived from impedance cardiography (Reyes del Paso et al., 2017).

## Conclusion

This study proposed a method for the characterization of prBR from the spontaneous variability of PR and DAP, fully homogeneous with the sequence approach typifying cBR from HP and SAP variability series, and tested it during incremental light-to-moderate bicycle ergometer exercise. The simultaneous characterization of both prBR and cBR provides a more insightful and complete description of one of the most important cardiovascular control reflexes (i.e., the BR). The relevance of the technique lies in the simultaneous, non-invasive, description of different components of the same control mechanism and from the complementary value of the extracted parameters as denoted by the degree of their correlation/uncorrelation and their different behaviors in response to the challenge. The approach might be fruitfully exploited in all pathological conditions featuring different degrees of responsiveness of regulatory mechanisms and/or mismatch among specific arms of regulatory reflexes such as in neurally-mediated syncope of different etiology. The abnormal increase of correlation between cBR and prBR markers, or their full uncoupling, or the lack of the observed trends with exercise might provide indications helpful to improve stratification of pathological populations even at the early signs of pathology.

## Author Contributions

AP analyzed the data and drafted the manuscript. MM, MP, and DL performed the experiments. AP, VB, BDM, BC, EV, MM, MP, and DL interpreted the data, revised the manuscript, and approved the final version of the manuscript.

## Conflict of Interest Statement

The authors declare that the research was conducted in the absence of any commercial or financial relationships that could be construed as a potential conflict of interest.

## Abbreviations

AP: arterial pressure
PR: peripheral resistances
BR: baroreflex
cBR: cardiac BR
prBR: BR of PR
BRS_cBR_: cBR sensitivity
BRS_prBR_: prBR sensitivity
DAP: diastolic
AP: E10, E20, E30, electronically braked bicycle ergometer exercise at 10, 20, and 30% of the maximum load
ECG: electrocardiogram
EI_cBR_: effectiveness index of the cBR
EI_prBR_: effectiveness index of the prBR
HP: heart period
MAP: mean AP
SBF: skin blood flow
MSBF: mean SBF
p: type I error probability
r: correlation coefficient
R: at rest in recumbent position on the bicycle ergometer
RC: recovery phase of the bicycle ergometer exercise
SAP: systolic AP
sBR: sympathetic BR
SEQ%_cBR_: percentage of cBR sequences
SEQ%_prBR_: percentage of prBR sequences
t_HP-SAP_: latency of the cBR
t_PR-DAP_: latency of the prBR
